# Advances in the Synthesis of Lignan Natural Products

**DOI:** 10.3390/molecules23123385

**Published:** 2018-12-19

**Authors:** Xianhe Fang, Xiangdong Hu

**Affiliations:** Key Laboratory of Synthetic and Natural Functional Molecule Chemistry of Ministry of Education, College of Chemistry & Materials Science, Northwest University, Xi’an 710127, China; xianhefang@stumail.nwu.edu.cn

**Keywords:** natural products, total synthesis, lignan

## Abstract

Lignans comprise a family of secondary metabolites existing widely in plants and also in human food sources. As important components, these compounds play remarkable roles in plants’ ecological functions as protection against herbivores and microorganisms. Meanwhile, foods rich in lignans have revealed potential to decrease of risk of cancers. To date, a number of promising bioactivities have been found for lignan natural products and their unnatural analogues, including antibacterial, antiviral, antitumor, antiplatelet, phosphodiesterase inhibition, 5-lipoxygenase inhibition, HIV reverse transcription inhibition, cytotoxic activities, antioxidant activities, immunosuppressive activities and antiasthmatic activities. Therefore, the synthesis of this family and also their analogues have attracted widespread interest from the synthetic organic chemistry community. Herein, we outline advances in the synthesis of lignan natural products in the last decade.

## 1. Introduction

Lignans are a family of secondary metabolites widely distributed in plants and human food sources. The story of lignans can traced back to 1942, when Harworth introduced the term for the first time to describe this family [1]. It is known that lignans have remarkable ecological functions in plants, providing protection against herbivores and microorganisms [2,3,4,5,6,7]. The consumption of foods rich in lignans has potential to decrease of risk of cancers [8,9,10,11]. During its long research history, this family has exhibited attractive pharmacological activities [12,13,14,15,16,17,18,19], such as antibacterial [20], antiviral [21,22,23,24], antitumor [25,26,27], antiplatelet [28,29], phosphodiesterase inhibition [30,31], 5-lipoxygenase inhibition [32,33,34], HIV reverse transcription inhibition [35,36,37], cytotoxic [38], antioxidant [39], immunosuppressive [40] and antiasthmatic properties [31].

Lignan compounds have dimeric structures formed through a β,β′-linkage between two phenylpropane units with different degrees of oxidation on the side-chain and variable substitution patterns on the phenyl ring. Traditionally, lignans are divided into two classes: classical lignans and neolignans. It should be noted that the term lignan in the literature refers to classical lignans in most cases. Regarding the classification of classical lignans, four different types are reported. The first one arranged classical lignans into three subgroups: acyclic lignan derivatives, arylnaphthalene derivatives and dibenzocyclooctadiene derivatives [41]. The second type includes six subgroups: dibenzylbutanes, dibenzylbutyrolactones, arylnaphthalenes, dibenzocyclooctadienes, substituted tetrahydrofurans and 2,6-diarylfurofurans [9,14]. The third one is comprised of eight subgroups: furofurans, furans, dibenzylbutanes, dibenzylbutyrolactones, aryltetralins, arylnaphthalenes, dibenzocyclooctadienes and dibenzylbutyrolactols [8,42,43,44,45]. The fourth one includes seven subgroups of lignan scaffolds: cyclobutanes, tetrahydrofurans, furofurans, dibenzylbutanes, aryltetralins, cycloheptenes and dibenzocyclooctadienes [46].

The synthesis of lignans and their analogues is an active field in the synthetic organic chemistry community. Tremendous synthetic efforts on this family have been well documented by reviews [9,10,11,12,13,14,41,47,48,49,50,51]. In recent years, several nice reviews have outlined progress of particular topics related to the synthesis of furofuran lignans [48], arylnaphthalene lactone analogues [47] and aryltetralin glycosides [49]. The present review will focus on the papers on the synthesis of lignans published from 2008–2018. In order to avoid unnecessary duplication, we will not discuss works already presented in previous reviews.

For the convenience of introduction of advances in the synthesis of lignans, we discuss three subgroups in present review, namely, acyclic lignan derivatives, dibenzocyclooctadiene derivatives and arylnaphthalene derivatives.

## 2. Advances in the Synthesis of Acyclic Lignan Derivatives

In the last decade, synthetic progress in acyclic lignan derivatives is related to lignans featuring dibenzyl tetrahydrofuran, dibenzylbutyrolactone, and diphenyltetrahydrofuranfurofuran skeletons.

**Scheme 1:**
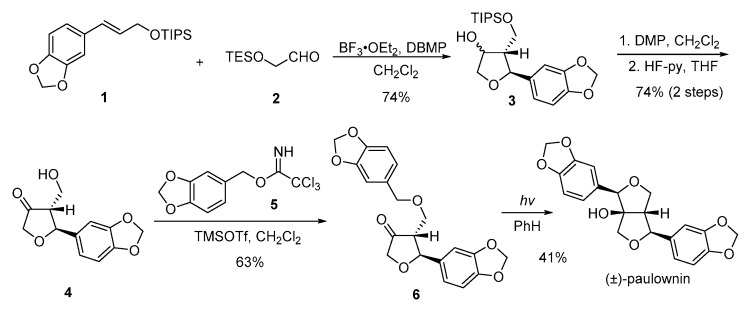
Synthesis of (±)-paulownin. Adapted from Angle et al. [52].

The synthesis of the acyclic lignan derivative (±)-paulownin (Scheme 1) was accomplished by Angle and coworkers in 2008 [52]. The key step is a formal [3 + 2]-cycloaddition between silyl ether **1** and aldehyde **2** in the presence of BF_3_OEt_2_ and 2,6-di-*tert*-butyl-4-methylphenol (DBMP), generating aryl tetrahydrofuran **3**. After oxidation and removal of the protecting group, the resulting product **4** was connected with imidate **5**, generating lactone **6**. The synthesis of (±)-paulownin was finished through photocyclization under a medium-pressure Hanovia lamp [53].

**Scheme 2:**
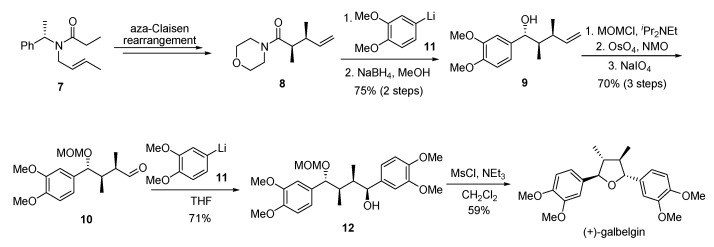
Synthesis of (+)-galbelgin. Adapted from Barker et al. [54].

In 2011, Barker and coworkers reported the total synthesis of (+)-galbelgin (Scheme 2) [54]. A stereoselective aza-Claisen rearrangement developed in their lab [55] afforded a reliable access to the original two stereocenters in chiral amide **8**. The subsequent nucleophilic addition from **11**, reduction, hydroxyl protection and double bond oxidative cleavage led to the formation of aldehyde **10**. The second nucleophilic addition from **11** afforded **12** with four adjacent stereocenters established. Methoxymethyl (MOM) group deprotection and cyclization completed the synthesis of (+)-galbelgin.

**Scheme 3:**
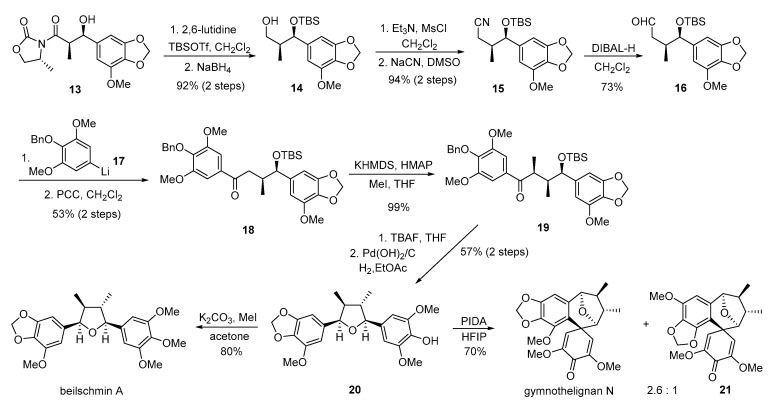
Synthesis of beilschmin A and gymnothelignan N. Adapted from She et al. [56].

She and coworkers reported the total synthesis of beilschmin A and gymnothelignan N in 2014 (Scheme 3) [56]. Alcohol **14** was prepared by hydroxyl protection of chiral amide **13** and subsequent reduction. Aldehyde **16** was obtained after homologation and reduction. The nucleophilic addition of **17** and oxidation afforded ketone **18**. Dibenzyl tetrahydrofurans **20** was obtained after a highly stereoselective introduction of a methyl group, deprotection and reduction. The synthesis of beilschmin A was finished after the methylation. Inspired by a biosynthetic proposal from She’s group, the challenging seven-membered ring skeleton in gymnothelignan N was constructed by an oxidative Friedel-Crafts reaction of compound **20** using phenyliodonium diacetate (PIDA) as the oxidant, finally affording gymnothelignan N.

**Scheme 4:**
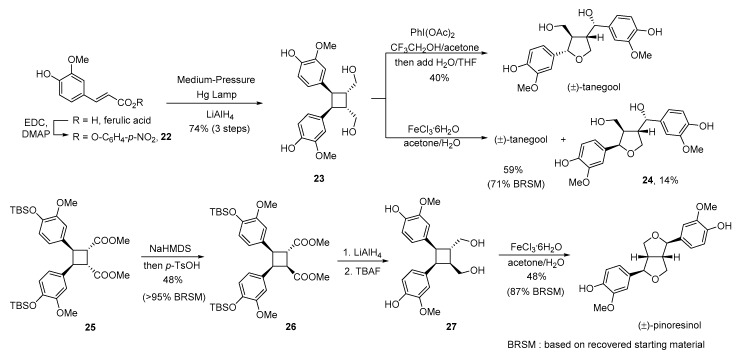
Bioinspired synthesis of (±)-tanegool and (±)-pinoresinol. Adapted from Lump et al. [46].

In 2015, Lump and coworkers reported a bioinspired total synthesis of (±)-tanegool and (±)-pinoresinol (Scheme 4) using [2 + 2] photodimerization and oxidative ring-opening as key steps [46]. The synthesis started with the esterification of ferulic acid. The resulting product **22** went through [2 + 2] photodimerization and reduction, generating diol **23** smoothly. The synthesis of (±)-tanegool through the expected oxidative ring-opening of **23** was achieved under different oxidative conditions. Moreover, synthesis of (±)-pinoresinol was also accomplished. Using the same strategy, *trans*-diester **26** was prepared from *cis*-diester **25**. Reduced product **27** was submitted to an oxidative ring-opening treatment using FeCl_3_·6H_2_O as the oxidant, finishing the synthesis of (±)-pinoresinol through an oxidative ring opening and two 5-*exo*-trig cyclization pathway.

**Scheme 5:**
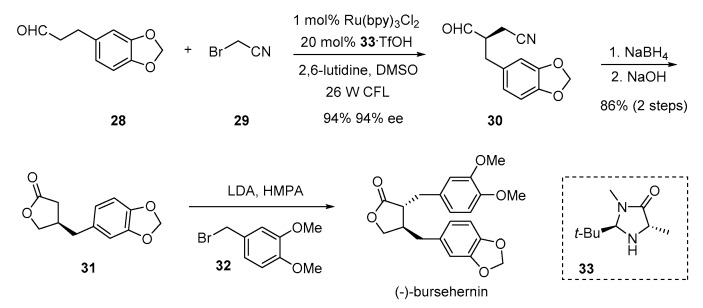
Asymmetric synthesis of (−)-bursehernin. Adapted from MacMillian et al. [60].

As powerful synthetic tools, photoredox-catalyzed tranformations have received considerable attention in recent decades [57,58,59]. In 2015, MacMillian and coworkers developed an enantioselective α-alkylation of aldehydes using a combination of photoredox catalysis and enamine catalysis and achieved the asymmetric synthesis of (−)-bursehernin through this strategy (Scheme 5) [60]. Using Ru(bpy)_3_Cl_2_, chiral amine **33** and a compact fluorescent lamp (CFL) light source, the α-alkylation of aldehyde **28** with bromonitrile **29** generated chiral aldehyde **30** in excellent yields and excellent enantioselectivity. Subsequent reduction and cyclization afforded lactone **31**. The synthesis of (−)-bursehernin was achieved by a highly stereoselective alkylation between **31** and bromide **32**.

**Scheme 6:**
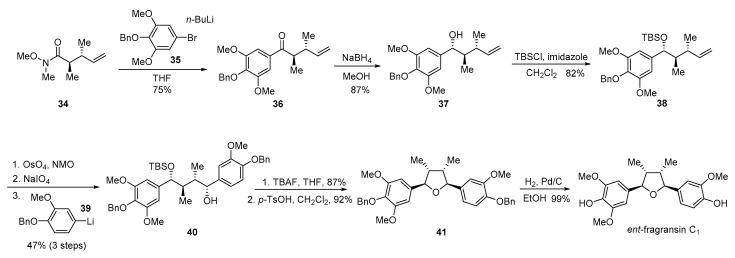
Asymmetric synthesis of *ent*-fragransin C_1_. Adapted from Soorukram et al. [61].

In 2017, Soorukram and coworkers reported the asymmetric synthesis of *ent*-fragransin C_1_ (Scheme 6) [61]. Ketone **36** was produced by the nucleophilic addition of the aryllithium species generated from **35** to chiral Weinreb amide **34**. The following stereoselective reduction led to the formation of alcohol **37**. After hydroxyl protection, double bond oxidative cleavage and nucleophilic addition from aryllithium **39**, compound **40** was furnished in good diastereoselectivity. Followed by the deprotection and cyclization treatments, the tetrahydrofuran ring in **41** was established. Finally, the synthesis of *ent*-fragransin C_1_ was accomplished through debenzylation under hydrogenation conditions.

**Scheme 7:**
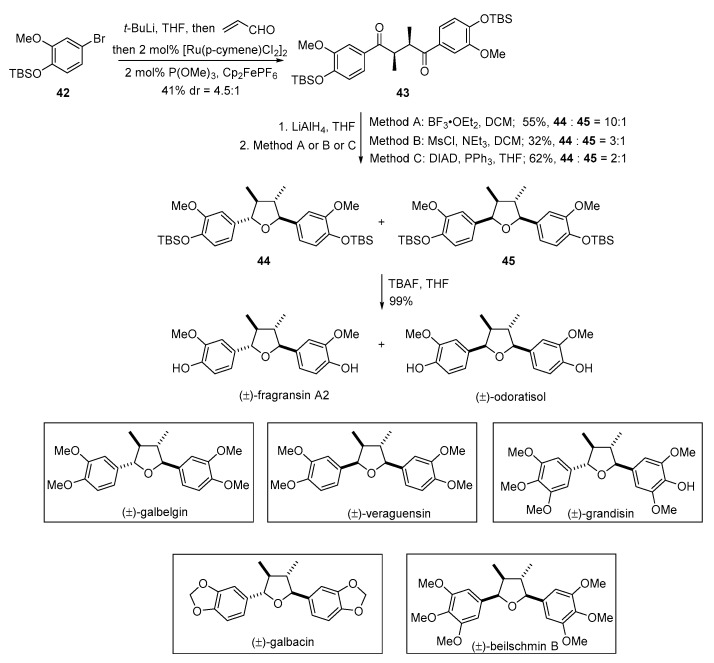
Bioinspired synthesis of seven acyclic lignans. Adapted from Jahn et al. [63].

Based on a tandem nucleophilic addition/Ru-catalyzed isomerization/SET oxidation/radical dimerization strategy [62], Jahn and coworkers reported a bioinspired total synthesis of multiple lignans in 2018 (Scheme 7) [63]. Using bromide **42** as the substrate, a smooth unprecedented tandem 1,2-nucleophilic addition/Ru-catalyzed isomerization/SET oxidation/radical dimerization afforded 1,4-diketone **43** with acceptable diastereoselectivity. After the reduction, three different treatments of **43** led to the formation of **44** and **45** in varied ratios. With the removal of double *t*-butyldimethylsilyl (TBS) protecting groups, the synthesis of (±)-fragransin A_2_ and (±)-odoratisol was achieved. Through the same strategy, Jahn and coworkers completed the synthesis of (±)-galbelgin, (±)-grandisin, (±)-galbacin, (±)-veraguensin, and (±)-beilschmin B.

**Scheme 8:**
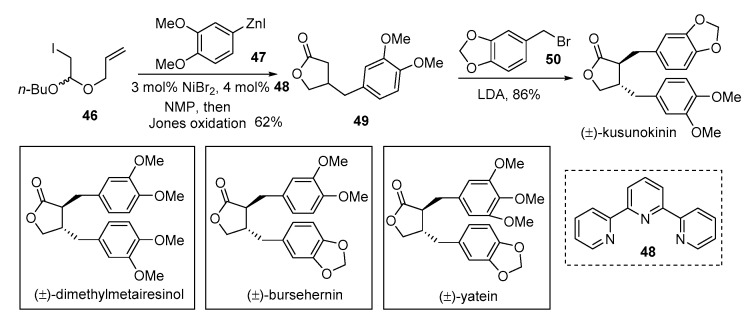
Synthesis of four acyclic lignans. Adapted from Giri et al. [64].

A Ni-catalyzed cyclization/cross-coupling strategy was developed and applied for the synthesis of (±)-kusunokinin, (±)-dimethylmetairesinol, (±)-bursehernin and (±)-yatein by Giri and coworkers in 2018 (Scheme 8) [64]. Ligand **48** was used for the Ni-catalyzed cyclization/cross-coupling between iodide **46** and aryl zinc reagent **47** followed by Jones oxidation, generating lactone **49** readily. Compound **49** was then connected with bromide **50** in a good diastereoselective manner, completing the synthesis of (±)-kusunokinin. The syntheses of (±)-dimethylmetairesinol, (±)-bursehernin and (±)-yatein were accomplished using the same protocol.

## 3. Advances in Synthesis of Dibenzocyclooctadiene Serivatives

Dibenzocyclooctadiene derivative lignans feature a particular eight-membered ring containing a chiral biaryl axis. Members of this subgroup possess various substitution patterns with two aryl rings and different stereocenters on the aliphatic bridge.

**Scheme 9:**
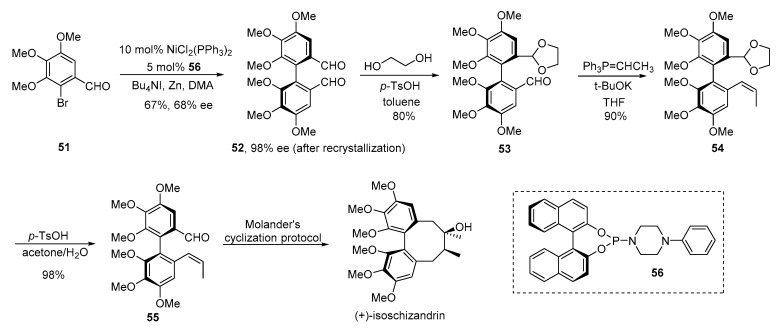
Asymmetric synthesis of (+)-isoschizandrin. Adapted from Lin and Xu et al. [65].

In 2010, an interesting Ni-catalyzed enantioselective Ullmann coupling of bis-*ortho*-substituted arylhalides was developed and applied to the asymmetric synthesis of (+)-isoschizandrin by Lin and coworkers (Scheme 9) [65]. With the application of chiral ligand **56**, the Ni-catalyzed enantioselective Ullmann coupling of bromide **51** gave the axial chiral biaryl dial **52** with acceptable enantioselectivity. Aldehyde **55** was prepared after monoprotection, Wittig reaction and deprotection operations. The synthesis of (+)-isoschizandrin was accomplished according to Molander’s cyclization protocol [66].

**Scheme 10:**
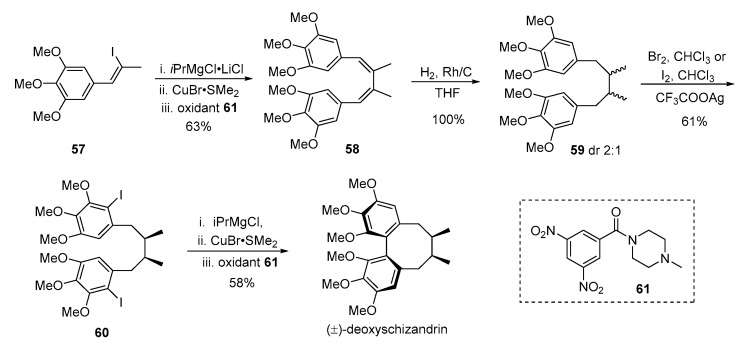
Synthesis of (±)-deoxyschizandrin. Adapted from Spring et al. [67].

Based on a double organocuprate oxidation strategy, Spring and coworkers reported the total synthesis of (±)-deoxyschizandrin in 2012 (Scheme 10) [67]. Symmetrical 1,3-diene **58** was prepared by the homo-coupling of alkenyl iodide **57** through a mild metalation, magnesio-cuprate transmetalation and subsequent oxidation using **61** as the oxidant [68]. Subsequent hydrogenation afforded **59** as a mixture of two diastereoisomers. After iodination, the expected iodide **60** was obtained. The synthesis of (±)-deoxyschizandrin was completed by an intramolecular organocuprate oxidation process, including metalation, magnesio-cuprate transmetalation and oxidation with **61**.

**Scheme 11:**
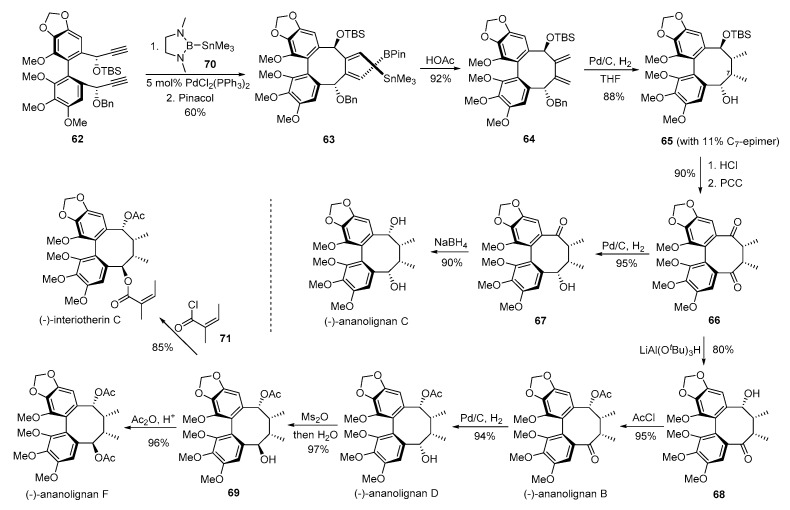
Synthesis of five dibenzocyclooctadienes lignans. Adapted from RajanBabu et al. [69].

In 2013, the RajanBabu group reported a general synthetic approach to multiple dibenzocyclooctadienes lignans via an interesting borostannylative cyclization (Scheme 11) [69]. In the presence of PdCl_2_(PPh_3_)_2_ and [B-Sn] reagent **70**, chiral diynyl precursor **62** was converted into dibenzocyclooctadiene **64** through a borostannylative cyclization and a subsequent acidification process [70].

The Subsequent hydrogenation afforded **65** as the major product. After the deprotection and oxidation, the general intermediate **66** was prepared. The synthesis of (−)-ananolignan C was achieved through two successive diastereoselective reductions of **66**. Meanwhile, the synthesis of (−)-ananolignan B was accomplished from the treatment of **66** with LiAl(O*^t^*Bu)_3_H and subsequent acetylation. The stereoselective hydrogenation of (−)-ananolignan B led to the formation of (−)-ananolignan D. The following configuration inversion of the hydroxyl group and actylation led to the synthesis of (−)-ananolignan F. In addition, (−)-interiotherin C can also be formed through the esterification of **69** and angeloyl chloride **71**.

**Scheme 12:**
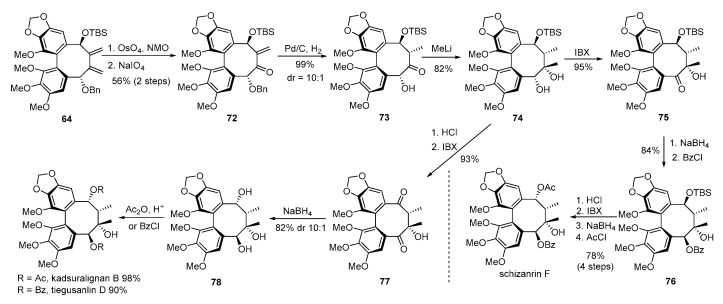
Synthesis of another three dibenzocyclooctadienes lignans. Adapted from RajanBabu et al. [69].

Synthesis of other three lignans was reported by RajanBabu and coworkers in the same paper (Scheme 12) [69]. Oxidative cleavage of the right-bottom double bond of **64** was applied for the formation of ketone **72**. Diol **74** was obtained from the debenzylation and methyllithium 1,2-addition of **72**. After hydroxyl oxidation, diastereoselective reduction and benzoyl protection steps, compound **76** was obtained. Starting from **76**, synthesis of schizanrin F was achieved by TBS deprotection, oxidation, diastereoselective reduction and acetylation process. Starting from diol **74** again, compound **78** can be prepared by TBS deprotection, oxidation and double diastereoselective reduction. Finally, the synthesis of kadasuralignan B and tiegusanlin D was accomplished through acetylation and benzoylation of **78**, respectively.

**Scheme 13:**
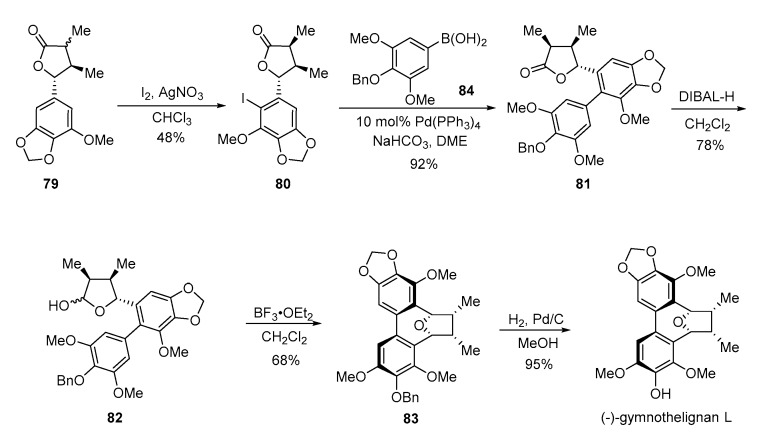
Bioinspired asymmetric synthesis of (-)-gymnothelignan L. Adapted from She et al. [71].

In 2018, a mild and asymmetric synthetic route to (−)-gymnothelignan L was developed by She and coworkers through a Suzuki-Miyaura coupling and a bioinspired desymmetric transannular Friedel-Crafts cyclization strategy (Scheme 13) [71]. Iodide **80** was obtained from iodination of compound **79**. The Suzuki-Miyaura coupling of **80** and arylboronic acid **84** formed biphenyl compound **81**, which was transformed into **82** using DIBAL-H as the reducing agent. Under acidic conditions, a bioinspired desymmetric transannular Friedel-Crafts cyclization of **82** occurred readily, generating **83**. After removal of the benzyl protecting group, the synthesis of (−)-gymnothelignan L was completed. At almost the same time, a similar strategy was applied in total synthesis (−)-gymnothelignan V by Soorukram and coworkers [72].

## 4. Advances in the Synthesis of Arylnaphthalene Derivatives

In the literature, the arylnaphthalene derivative lignan subgroup includes arylnaphthalenes and aryltetralins. Structurally, these lignans have a substituted naphthalene core. It should be mentioned that, due to their excellent biological characters, several clinically used antitumor drugs are derived from the well-known member, podophyllotoxin, and its glycosides [49].

**Scheme 14:**
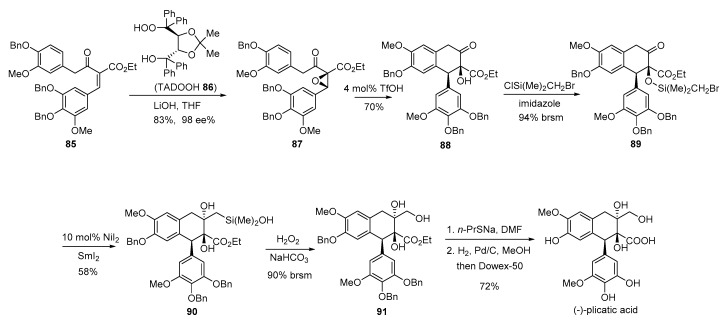
Asymmetric synthesis of (−)-plicatic acid. Adapted from Deng et al. [73].

In 2009, Deng and coworkers reported an asymmetric total synthesis of (−)-plicatic acid (Scheme 14) [73]. The enantioselective epoxidation of trisubstitued olefin **85** was applied for the introduction of the original chiral stereocenters. Excellent enantioselectivity was obtained from the application of chiral (*S*,*S*)-TADOOH **86**. Intramolecular Friedel-Crafts reaction of **87** formed the six-membered ring of **88** effectively. Subsequent silylation and intramolecular Barbier reaction under SmI_2_/NiI_2_ conditions afforded a diastereoselective access to diol **90**. After Fleming-Tamao- Kumada oxidation [74,75], **91** was furnished. The synthesis of (−)-plicatic acid was completed following hydration and global debenzylation.

**Scheme 15:**
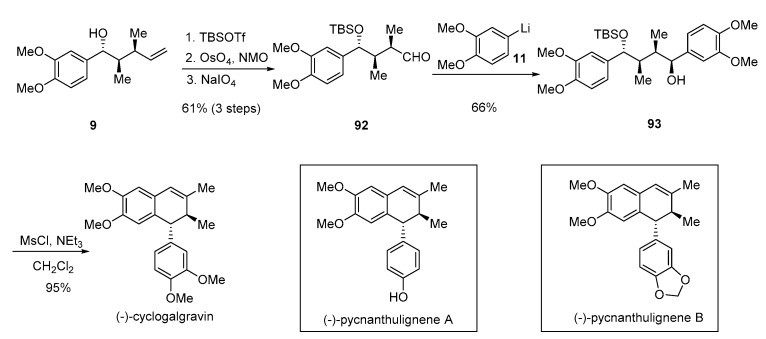
Asymmetric synthesis of (−)-cyclogalgravin. Adapted from Barker et al. [54].

The stereoselective aza-Claisen rearrangement strategy developed by Barker and coworkers was not only effective for asymmetric synthesis of (+)-galbelgin (Scheme 2), but also for asymmetric synthesis of (−)-cyclogalgravin, and (−)-pycnanthulignenes A and B (Scheme 15) [54]. Through the aza-Claisen rearrangement strategy, alcohol **9** (Scheme 2) was prepared and submitted to hydroxyl protection and double bond oxidation, generating aldehyde **92**. The addition from aryllithium **11** gave compound **93**. The synthesis of (−)-cyclogalgravin was achieved through cyclization. Employing same protocol, Barker’s group also finished an asymmetric synthesis of (−)-pycnanthulignenes A and B.

**Scheme 16:**
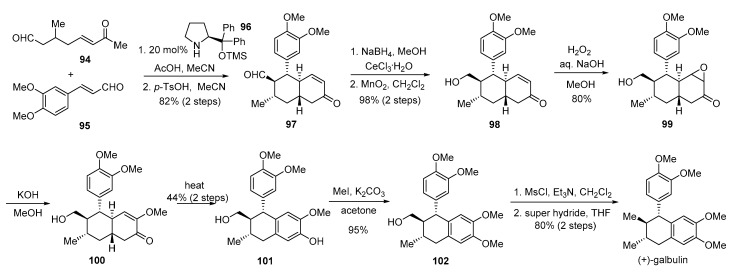
Enantioselective synthesis of (+)-galbulin. Adapted from Hong et al. [76].

In 2012, Hong and coworkers reported the enantioselective total synthesis of (+)-galbulin using an organocatalytic asymmetric Michael-Michael-aldol cascade (Scheme 16) [76]. Under the promotion of Jørgensen-Hayashi catalyst **96**, ketoaldehyde **97** was readily prepared from the asymmetric Michael-Michael-aldol cascade of **94** and **95**. Compound **99** was produced by reduction, oxidation and epoxidation treatments of **97**. Following epoxide ring-opening and aromatization, compound **101** was obtained. The synthesis of (+)-galbulin was finally accomplished through selective methylation and dehydroxylation processes.

**Scheme 17:**
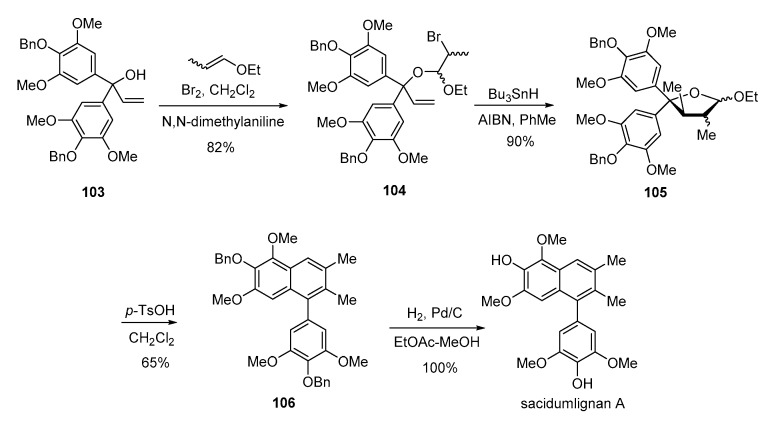
Synthesis of sacidumlignan A. Adapted from Peng et al. [77].

Peng and coworkers reported in 2013 the synthesis of sacidumlignan A employing Ueno-Stork radical cyclization and skeletal rearrangement strategy (Scheme 17) [77]. Alcohol **103** was connected with ethyl propenyl ether in the presence of bromine, readily generating **104**. The Ueno-Stork radical cyclization of **104** was enabled by Bu_3_SnH and AIBN.

The resulting **105** was submitted to a skeletal rearrangement, affording arylnaphthalene **106**. The synthesis of sacidumlignan A was achieved after benzyl deprotection.

**Scheme 18:**
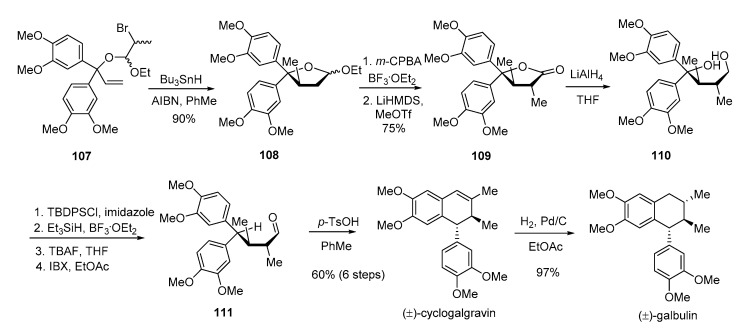
Synthesis of (±)-cyclogalgravin and (±)-galbulin. Adapted from Peng et al. [78].

The same year, Peng and coworkers also reported the total synthesis of (±)-cyclogalgravin and (±)-galbulin (Scheme 18) [78]. Cyclic acetal **108** was obtained from Ueno-Stork radical cyclization of **107**. Diol **110** was prepared by an oxidation, methylation and reduction process. Subsequent selective hydroxyl protection, dehydroxylation, deprotection and oxidation led to the generation of aldehyde **111**. Next an intramolecular Friedel-Crafts reaction was applied for the synthesis of (±)-cyclogalgravin. The synthesis of (±)-galbulin was readily achieved by the stereoselective hydrogenation of (±)-cyclogalgravin.

**Scheme 19:**
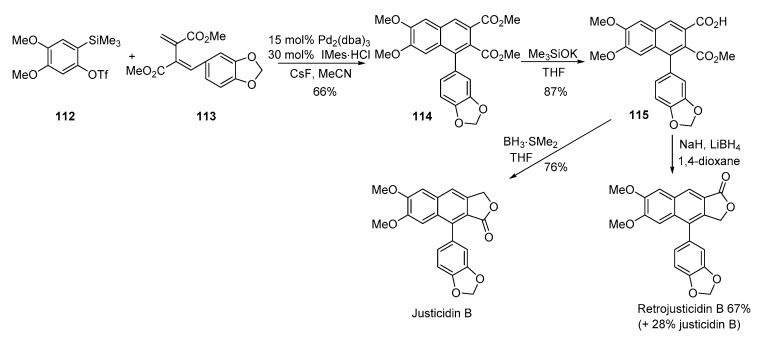
Synthesis of justicidin B and retrojusticiding B. Adapted from Argade et al. [79].

In 2013, Argade and coworkers reported a novel strategy to construct arylnaphthalene frameworks via Pd-promoted [2 + 2 + 2] cyclization (Scheme 19), which enabled the synthesis of justicidin B and retrojusticiding B [79]. Through a Pd-promoted [2 + 2 + 2] cyclization process, aryne precursor **112** was connected with diene **113**, generating arylnaphthalene **114**. After the regioselective hydrolysis of the ester group, the synthesis of justicidin B was achieved through a chemoselective reduction of the acid group using BH_3_·SMe_2_ and subsequent lactonization. Meanwhile, the synthesis of retrojusticidin B was achieved through the reduction of **115** and subsequent lactonization.

**Scheme 20:**
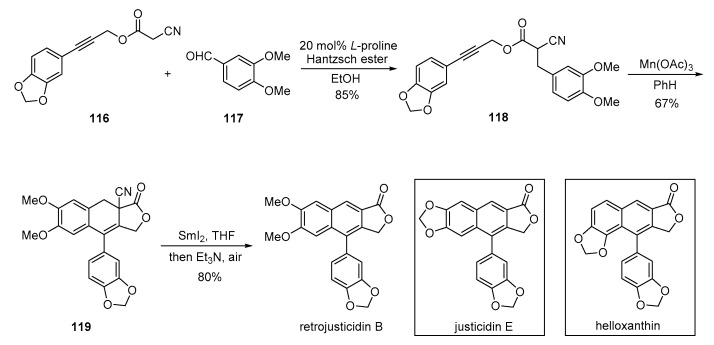
Synthesis of arylnaphthalene derivative lignans. Adapted from Shia et al., 2015 [80].

Shia and coworkers reported in 2015 the synthesis of three arylnaphthalene derivative lignans using a Mn(III)-mediated free radical cyclization cascade (Scheme 20) [80]. Knoevenagel condensation of α-cyano ester **116** with aldehyde **117** and subsequent reduction was applied for the generation of α-cyano ester **118**. The following oxidative free radical cyclization cascade enabled by Mn(OAc)_3_ afforded access to compound **119**. The synthesis of retrojusticidin B was accomplished after decyanation and aromatization operations. Using the same strategy, the synthesis of justicidin E and helioxanthin was completed.

**Scheme 21:**
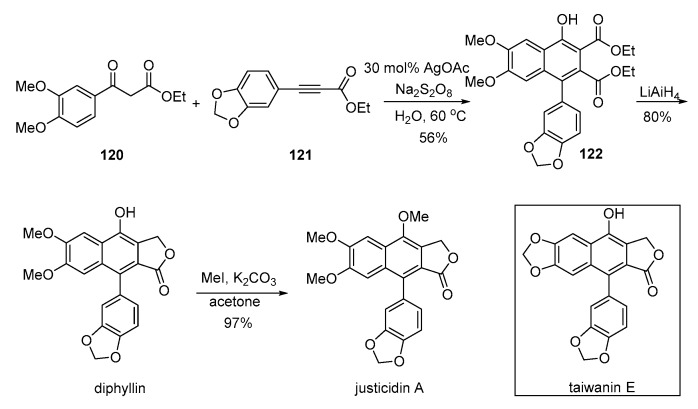
Synthesis of arylnaphthalene lignans. Adapted from Narender et al. [81].

In 2015, Narender and coworkers reported an interesting Ag-promoted radical addition/cyclization process for the construction of highly substituted α-naphthol skeletons (Scheme 21) and the synthesis of three arylnaphthalene lignans [81]. Through the Ag-promoted radical addition/cyclization between ketoester **120** and aryl propiolate **121**, polysubstituted arynaphthol **122** was readily prepared. The synthesis of diphyllin was finished under known reductive-lactonization conditions [82]. The synthesis of justicidin A was then achieved through methylation of diphyllin. The synthesis of taiwanin E was also accomplished.

**Scheme 22:**
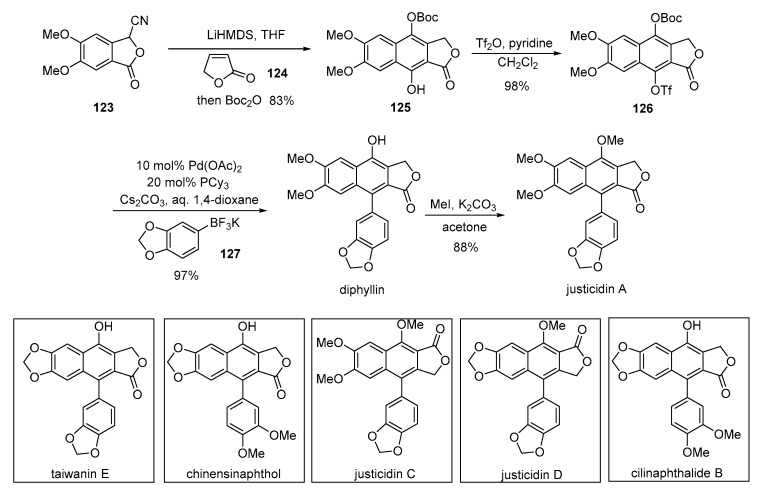
Synthesis of seven arylnaphthalene lignans. Adapted from Ham et al. [83].

In 2017 Ham and coworkers reported the synthesis of seven arylnaphthalene derivative lignans based on a strategy involving Hauser-Kraus annulation and Suzuki-Miyaura cross-coupling (Scheme 22) [83]. In the presence of LiHMDS, the Hauser-Kraus annulation between cyanophthalide **123** and γ-crotonolactone **124** and subsequent protection treatment gave arylnaphthalene **125**. The synthesis of diphllin was finished by the subsequent Suzuki-Miyaura cross-coupling of **126** and potassium aryltrifluoroborate **127**. Justicidin A was produced by the methylation of diphllin. The syntheses of taiwannin E, chinensinaphthol justicidin C, justicidin D and cilinaphthalide B were also finished.

**Scheme 23:**
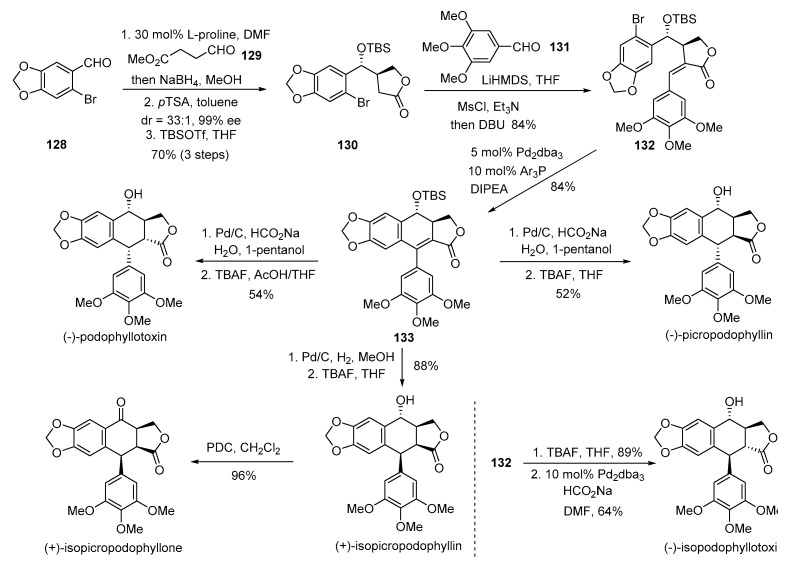
Synthesis of (−)-podophyllotoxin and three natural analogues. Adapted from Hajra et al. [84].

Hajra and coworkers reported the enantioselective total synthesis of (−)-podophyllotoxin and natural analogues in 2017 (Scheme 23) [84]. The L-proline-catalyzed asymmetric cross aldol reaction between 6-bromopiperonal **128** and aldehyde **129** introduced original stereocenters with excellent diastereoselectivity and excellent stereoselectivity at gram scale. Lactone **130** was obtained after reduction, lactonization and TBS protection operations. *Z*-Benzylidene lactone **132** was prepared through an aldol reaction between **130** and aldehyde **131**, and subsequent elimination. The intramolecular Heck reaction between trisubstituted *Z*-alkene motif and the bulky bromoarene motif in **132** happened smoothly, generating compound **133** in good yields. Notably, under different hydrogenation conditions, three stereoselective pathways of **133** led to the synthesis of (−)-podophyllotoxin, (−)-picropodophyllin, (+)-isopicropodophyllin, respectively. Meanwhile, the synthesis of (+)-isopicropodophyllone was achieved through the oxidation of (+)-isopicropodophyllin. The synthesis of (−)-isopodophyllotoxin can be accomplished through a TBS deprotection and reductive Heck reaction process from **132**.

**Scheme 24:**
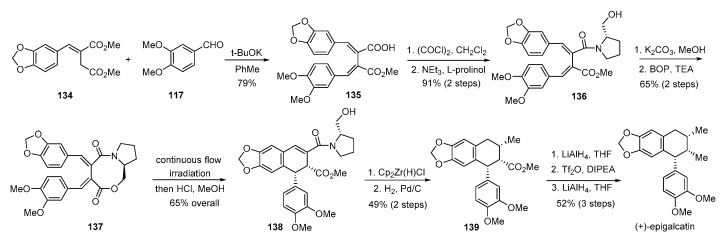
Synthesis of (+)-epigalcatin. Adapted from Czarnocki et al. [85].

Czarnocki and coworkers reported in 2018 the total synthesis of (+)-epigalcatin using a photocyclization process under continuous flow UV irradiation conditions (Scheme 24) [85]. Diester **134** was condensed with aldehyde **120** at basic conditions, affording *E*,*E*-bisbenzylidenesuccinic acid **135**. Through an amidation process, L-prolinol was introduced in amide **136** as a chiral auxiliary. Eight-membered ring compound **137** was prepared via following hydrolysis and macrolactonization. Under continuous flow irradiation with UV light, the photocyclization of **137** furnished **138** smoothly [86]. Remove of the chiral auxiliary, hydrogenation of the double bond and simultaneous reduction of formyl group led to the formation of ester **139**. The synthesis of (+)-epigalcatin was achieved through subsequent reductive transformations of the methyl ester of **139** into a methyl group via three steps.

**Scheme 25:**
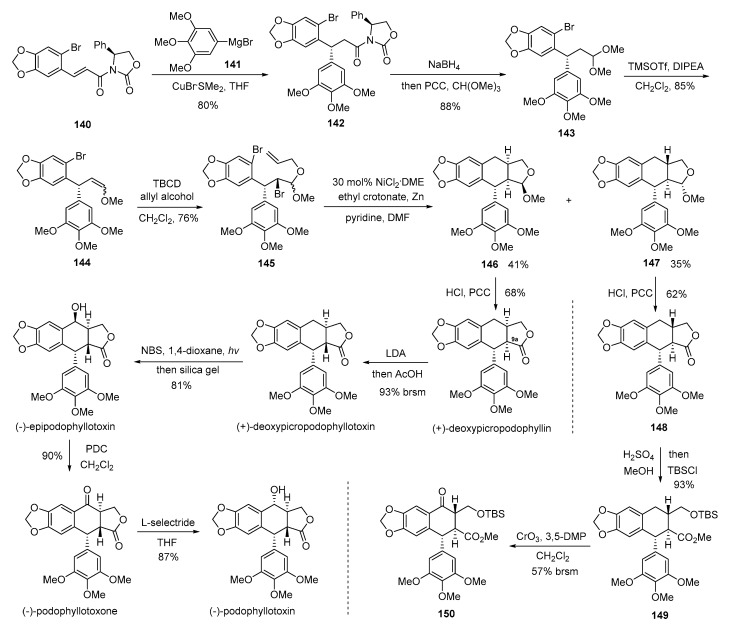
Synthesis of (−)-podophyllotoxin and four natural analogues. Adapted from Peng et al. [87].

Peng and coworkers reported the total synthesis of (−)-podophyllotoxin and four natural analogues using a Ni-catalyzed reductive cascade in 2018 (Scheme 25) [87]. The asymmetric conjugated addition of **141** to chiral α,β-unsaturated amide **140** introduced the first stereocenter. Enol ether **144** was obtained after subsequent reduction, oxidation, acetal formation and elimination. β-Bromoacetal **145** was produced using 2,4,4,6-tetrabromo-2,5-cyclohexadienone (TBCD). With the application of the Ni-catalyzed reductive cascade [88], both **146** and **147** were produced in moderate yields. After the hydration and oxidation of **146**, the synthesis of (+)-deoxypicropodophyllin was accomplished. (+)-Isodeocypodophyllotoxin can be synthesized from the epimerization at C9a of (+)-deoxypicropodophyllin under basic conditions. With the radical bromination under visible-light irradiation [89], and further oxidation treatments, the synthesis of (−)-epipodophyllotoxin and (−)-podophyllotoxone was achieved in a stepwise fashion. The stereoselective reduction of (−)-podophyllotoxone using l-Selectride gave (−)-podophyllotoxin. Additionally, compound **147** can be transformed into Meyer’s **150** intermediate for synthesis of (−)-picropodophyllin and (−)-picropodophyllone through three regular operations [90].

**Scheme 26:**
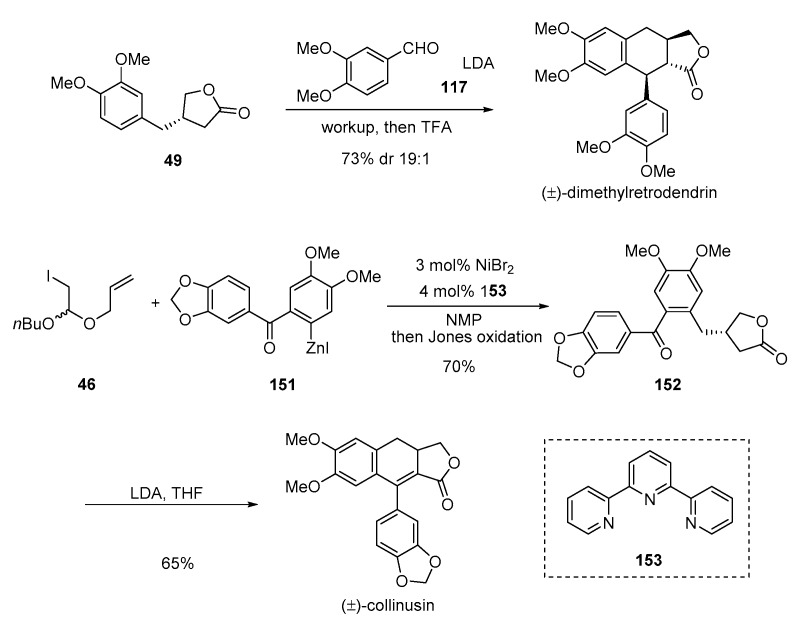
Synthesis of (±)-dimethylretrodendrin and (±)-collinusin. Adapted from Giri et al. [64].

The Ni-catalyzed cyclization/cross-coupling has been verified as a suitable strategy for not only the synthesis of multiple acyclic lignan derivatives (Scheme 8) but also the synthesis of (±)-dimethylretrodendrin and (±)-collinusin (Scheme 26) by Giri and coworker [64]. Lactone **49** was obtanied from the Ni-catalyzed cyclization/cross-coupling process (Scheme 8). The stereoselective aldol reaction between **49** and aldehyde **117** and following intramolecular Friedel-Crafts reaction led to the formation of (±)-dimethylretrodendrin.

In the presence of **153**, the Ni-catalyzed cyclization/cross-coupling of **46** and **151** and following oxidation gave lactone **152**. The synthesis of (±)-collinusin was completed by subsequent intramolecular nucleophilic addition and dehydration.

**Scheme 27:**
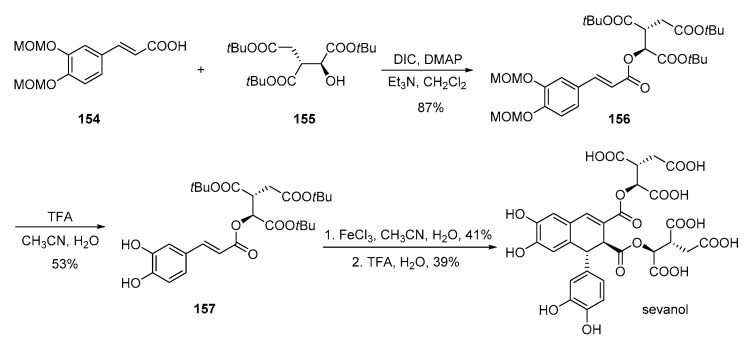
Synthesis of sevanol. Adapted from Belozerova et al. [91].

In 2018, Belozerova and coworkers reported the synthesis of sevanol via an oxidative dimerization strategy (Scheme 27) [91]. Chiral ester **156** was prepared by esterification of acid **154** and chiral alcohol **155**. After the removal of two MOM protecting groups of **156**, compound **157** was submitted to FeCl_3_-promoted oxidative dimerization, affording sevanol after the hydrolysis of all three ester groups.

**Scheme 28:**
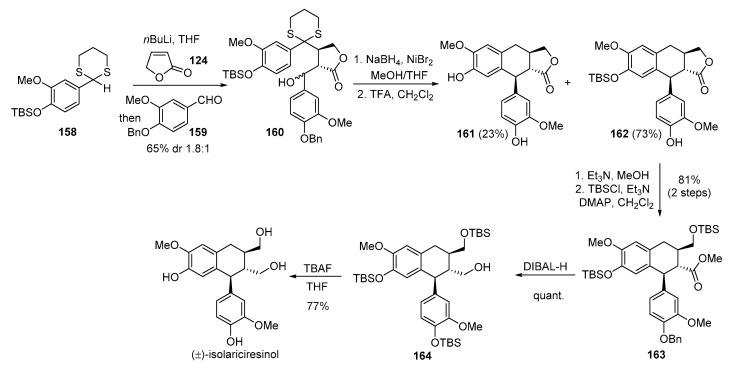
Synthesis of (±)-isolariciresinol. Adapted from Aria et al. [92].

Aria and coworkers reported total synthesis of (±)-isolariciresinol using a tandem Michael-aldol reaction in 2018 (Scheme 28) [92]. Alcohol **160** was obtained as a diastereomeric mixture from the tandem Michael-aldol reaction of dithiane **158**, lactone **124** and aldehyde **159** under basic conditions. After the cleavage of the dithiane substituent and TBS, the following cyclization furnished **162** as the major product.

Ester **163** was prepared from methanolysis of the lactone ring and TBS protection operations. The synthesis of (±)-isolariciresinol was achieved through the subsequent reduction and deprotection treatments.

**Scheme 29:**
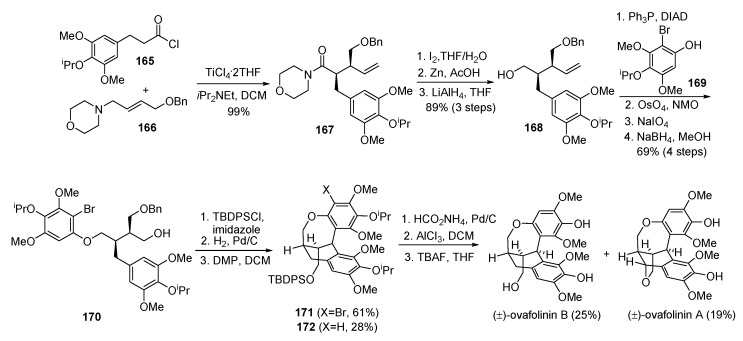
Synthesis of (±)-ovafolinins A and B. Adapted from Barker et al. [93].

Barker and coworkers reported in 2017 the first total synthesis of (±)-ovafolinins A and B through the acyl-Claisen rearrangement developed in their lab and a cascade cyclization enabled by bulky protecting groups (Scheme 29) [93]. Notably, (±)-ovafolinins A and B have polycyclic skeletons rarely found in lignans. The acyl-Claisen rearrangement between acid chloride **165** and allylic morpholine **166** afforded amide **167** as a single diastereoisomer and in excellent yields. Alcohol **168** was prepared by hydration and reduction. Phenol **169** was introduced through a Mitsunobu reaction. Alcohol **170** was obtained after the oxidative cleavage of the double bond and following reduction. The subsequent *t*-butyldiphenylsilyl (TBDPS) protection, debenzylation and oxidation led to the formation of compounds **171** and **172** through a cascade cyclization enabled by the TBDPS group. The synthesis of (±)-ovafolinins A and B was achieved after subsequent hydrogenation and deprotection.

**Scheme 30:**
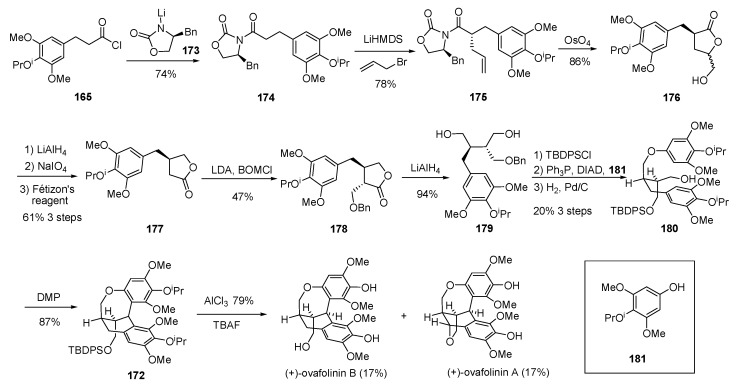
Asymmetric synthesis of (+)-ovafolinins A and B. Adapted from Barker et al. [93].

Taking advantage of the above achievement, Barker and coworkers reported the first asymmetric total synthesis of (+)-ovafolinins A and B (Scheme 30). Starting from acid chloride **165** again, chiral amide **174** was first prepared. Stereoselective allylation and dihydroxylation of the double bond led to the generation of lactone **176**. After the reduction and oxidative cleavage of the 1,2-diol motif, lactone **177** was formed by Fétizon oxidation. The introduction of the benzyloxymethyl group and the following reduction led to the formation of **179**. After TBDPS protection, Mitsunobu reaction with **181** and debenzylation, alcohol **180** was obtained. Chiral **172** was formed through a cascade cyclization under oxidative conditions. Finally, the first asymmetric synthesis of (+)-ovafolinins A and B was achieved after deprotection operations. Based on optical rotation comparisons between the synthetic samples and the natural compounds, Barker’s group demonstrated that natural ovafolinins A and B were both isolated in scalemic mixtures. And the original stereochemical assignment of natural ovafolinin B was corrected.

**Scheme 31:**
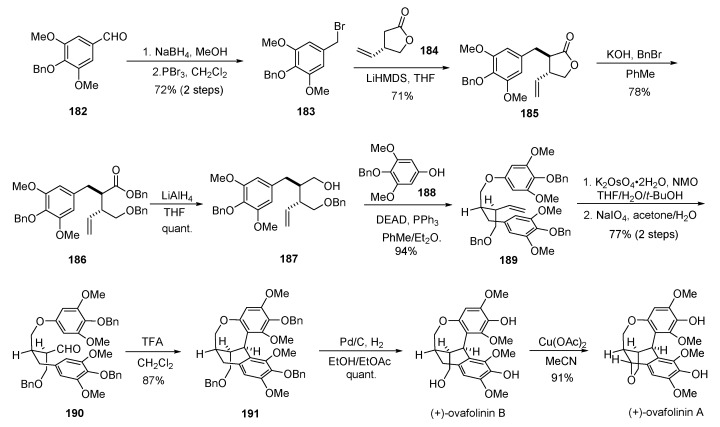
Asymmetric synthesis of (+)-ovafolinins A and B. Adapted from Hu et al., 2018 [94].

Recently, we developed a new asymmetric synthetic route to (+)-ovafolinins A and B (Scheme 31) [94]. Starting from benzyl syringaldehyde **182**, bromide **183** was prepared after reduction and bromination. The diastereoselective alkylation of (*S*)-Taniguchi lactone **184** introduced two adjacent stereogenic centers in excellent stereoselectivity, affording lactone **185**. Subsequent double benzyl protection opened the lactone ring and generated ester **186**. Compound **189** was obtained from the reduction and connected with **188** through Mitsunobu reaction. After oxidative cleavage of double bond, aldehyde **190** was obtained. The polycyclic skeleton in **191** was constructed through a double Friedel-Crafts reaction of **190**. The synthesis of (+)-ovafolinin B was accomplished through the global debenzylation. And the synthesis of (+)-ovafolinin A was achieved through subsequent benzylic oxidation cyclization enabled by Cu(OAc)_2_.

## 5. Conclusions

In this review, we have summarized the advances in the synthesis of lignan natural products reported from 2008 to 2018. Synthetic progress in three areas was outlined: acyclic lignan derivatives, dibenzocycooctadiene derivative and arylnaphthalene derivatives. Novel synthetic methodologies had been applied for construction of challenging structures existing in lignan natural products. As the result, many elegant synthetic approaches to lignans had been developed. However, as a long term program, the promising biological features and development of concise synthetic approaches to lignan natural products and their analogues are continuing to attract more and more interest from the pharmaceutical industry and the organic synthesis community.
